# Supplementary material for: Widening the landscape of transcriptional regulation of green algal photoprotection
Source: Nat Commun. 2023 May 10;14:2687. doi: 10.1038/s41467-023-38183-4 (PMC10172295; doi:10.1038/s41467-023-38183-4)
Supplement: Supplementary file 1 — Supplementary Information [file 41467_2023_38183_MOESM1_ESM.pdf]

Supplementary Information for

Widening the landscape of transcriptional regulation of green algal photoprotection

Authors

Marius Arend[†], Yizhong Yuan[†], M. Águila Ruiz-Sola, Nooshin Omranian, Zoran Nikoloski[*], Dimitris Petroutsos[*]

[*]Corresponding Authors. nikoloski@mpimp-golm.mpg.de (Z.N.), dimitris.petroutsos@cea.fr (D.P.)

This PDF file includes:

Supplementary Notes 1 and 2

Supplementary Figs. 1 to 15

Other Supplementary Materials for this manuscript include the following:

Supplementary Tables 1 to 13 (to be downloaded as a single excel file)

Supplementary Note 1

In the course of independent projects aiming to identify novel regulators of *LHCSR3*, we have investigated the role of four transcriptional factors, designated as TF1-4, in regulating expression of *LHCSR3.1* (*TF1*: MYB-like DNA-binding protein, Cre01.g034350; *TF2*: RWP8, RWP-RK transcription factor, Cre04.g218050; *TF3*: RWP5, RWP-RK transcription factor, Cre06.g285600; *TF4*: bHLH domain-containing protein, Cre07.g349152). Mutants bearing mutations in TF1-4 genes were ordered from the CliP library [1]. After confirming (Supplementary Fig. 2a) that expression of the genes of interest was abolished (*tf1-3* mutants) or was significantly higher than in WT (for the case of *tf4-oe*), we applied the following experimental setup to probe for *LHCSR3.1* expression: WT and mutant cells were acclimated for 16h in LL (15 µmol photons m^{-2} s^{-1}). After sampling under the LL condition, light intensity was increased to 300 µmol photons m^{-2} s^{-1} (HL); samples for RNA extraction were taken after 1 h of exposure to HL. Our data showed that WT and mutants had similar expression levels of *LHCSR3.1* (**Supplementary Fig. 2**), excluding a role of these TFs in regulating transcription of *LHCSR3.1*.

Supplementary Note 2

Simulation studies[2] indicated that the top 10% of the edges in the consensus network are enriched in positive interactions if the underlying approaches perform better than guessing. Thus, a higher overlap with the consensus networks is expected to result in improved predictions. We found that the interactions from the network deconvolution approach exhibited the largest overlap with the consensus network, while GGM assigns the highest rank to consensus interactions (**Supplementary Fig. 4a**). Repeating this analysis by including the two worse performing approaches (i.e. ARACNE and Silencing) resulted in no qualitative changes in the overlap (**Supplementary Fig. 4b**) and only 7.47% of difference in the included interactions compared to the original consensus network, demonstrating the robustness of the inferred interactions. Considering the 10% network density threshold, we also inspected the proportion of TF-TF interactions in the different networks and their consensus; we found that it ranges from 2.55% in the GENIE3 network to 2.77% in the network deconvolution approach (**Supplementary Fig. 4c**). Since the fully connected network, containing all possible TF-TF and TF-target interactions, has a relative TF-TF interaction content of 2.52%, these findings suggest an enrichment of TF-TF interactions in GRNs inferred by all five approaches considered in the consensus.

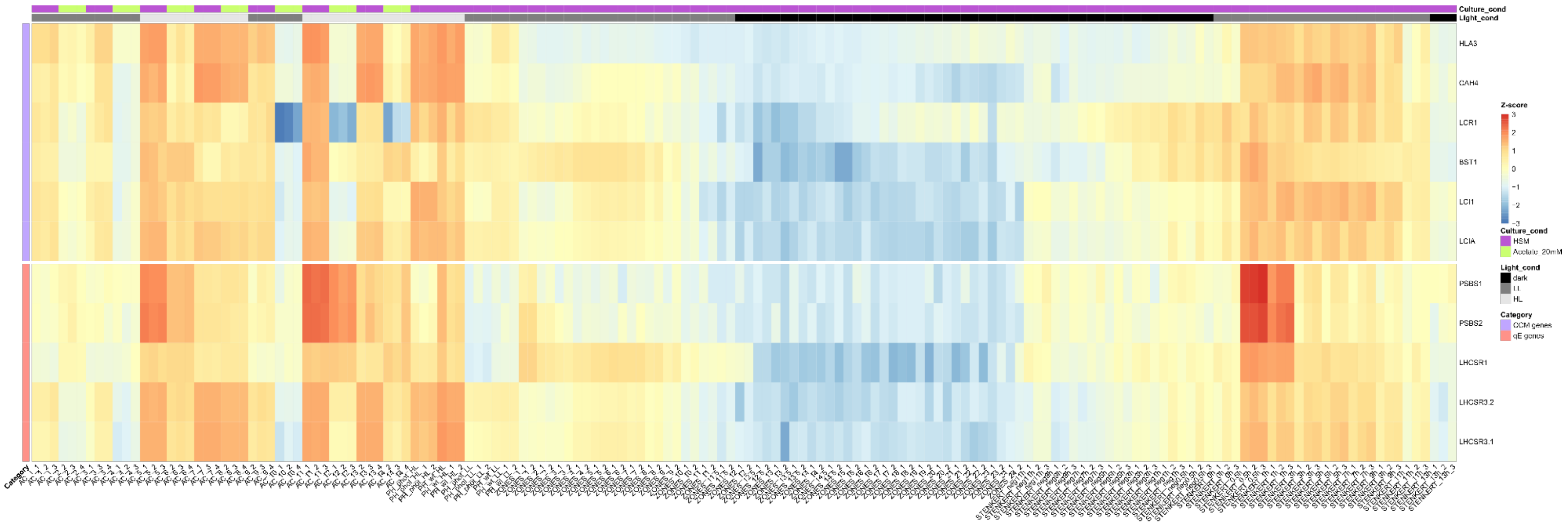

Supplementary Fig. 1: Z-score of log-transcript levels for representative CCM and qE genes in the used RNAseq data set. Enlarged representation of **Fig. 1a** with sample names included (see **Supplementary Table 1** for more details). Expression levels of representative carbon concentrating mechanism *(CCM)* and qE genes are plotted over all samples used for network inference (z-scaled log values are depicted*,* high values red, low values blue). The column annotation gives information on the culture conditions and data set (purple – Sueoka's high salt medium (HSM), green – HSM + 20mM acetate, light grey – high light (HL), dark grey - low light (LL), black – no light) see also **Supplementary Table 1**); no clustering was applied. The last digit of the column names indicates the replicate number.

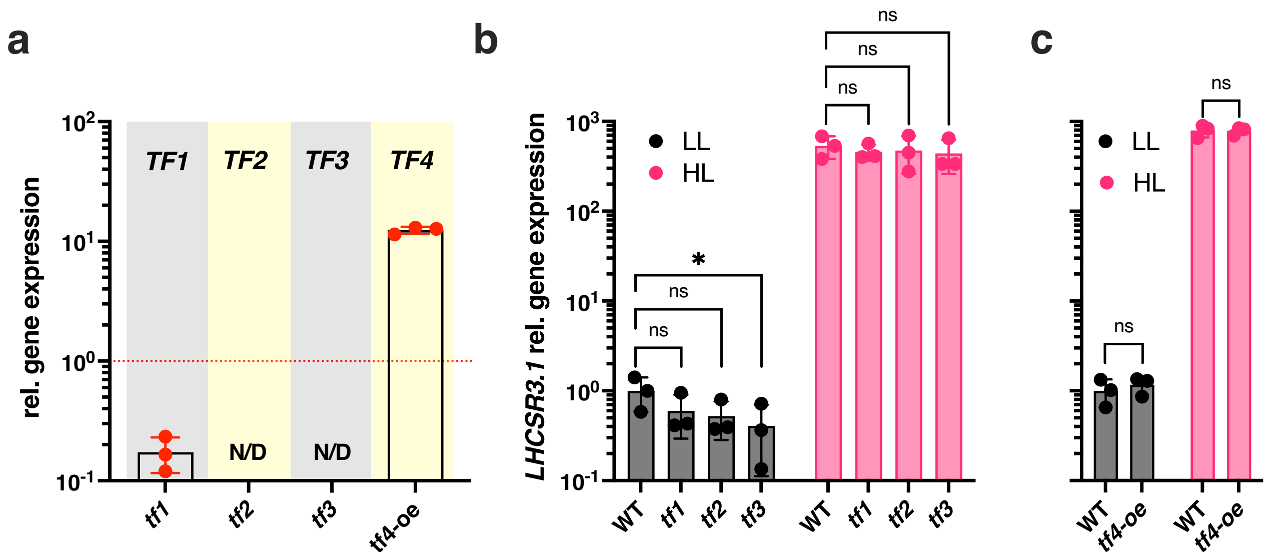

Supplementary Fig. 2: *LHCSR3.1* expression in mutants bearing mutations in transcription factors TF1-4. a Relative expression levels of *TF1-4 in tf1, tf2, tf3* and *tf4* mutant strains, grown under LL conditions in tris-acetate-phosphate (TAP) medium, normalized to WT (indicated as a red dashed line in the figure); N/D: non-detectable (*n* = 3 biological samples, mean ± sd). **b** and **c** WT, *tf1, tf2, tf3 and tf4* cells were acclimated for 16h in LL (15 µmol photons m^{-2} s^{-1}). After sampling under the LL conditions, light intensity was increased to 300 µmol photons m^{-2} s^{-1} (HL); samples were taken 1 h after exposure to HL. Shown are relative expression levels of *LHCSR3.1* at the indicated conditions normalized to WT LL (*n* = 3 biological samples, mean ± sd) for **b** WT (CC-4533) and *tf1-3* and **c** for WT (CC-125) and *tf4-oe*, a strain overexpressing *tf4* in the CC-125 background. The p-values for the comparisons between the mutants and the WT are based on ANOVA Dunnett's multiple comparisons test using log10- transformed values; the p-values are indicated in the graphs (*, $P < 0.005$; ns: non-significant). *TF1*: MYB-like DNA-binding protein, Cre01.g034350; *TF2*: RWP8, RWP-RK transcription factor, Cre04.g218050; *TF3*: RWP5, RWP-RK transcription factor, Cre06.g285600; *TF4*: bHLH domain-containing protein, Cre07.g349152).

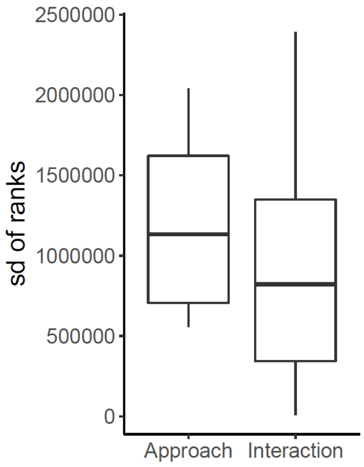

Supplementary Fig. 3: Variation in ranks of known (positive) interactions across approaches and interactions from different inference approaches. Boxplots of the standard deviation of ranks calculated for the curated interactions plotted in **Fig. 1b** over all ranks assigned by one approach (n=5 independent network inference approaches) or over all ranks assigned to one interaction (n=18 known interactions). The boxes mark the 2nd (25th percentile) and 3rd quartile (75th percentile), with the line in the middle marking the median (50th percentile). Whiskers extend to the most extreme measurement whose difference to the median is less than 1.5 interquartile ranges.

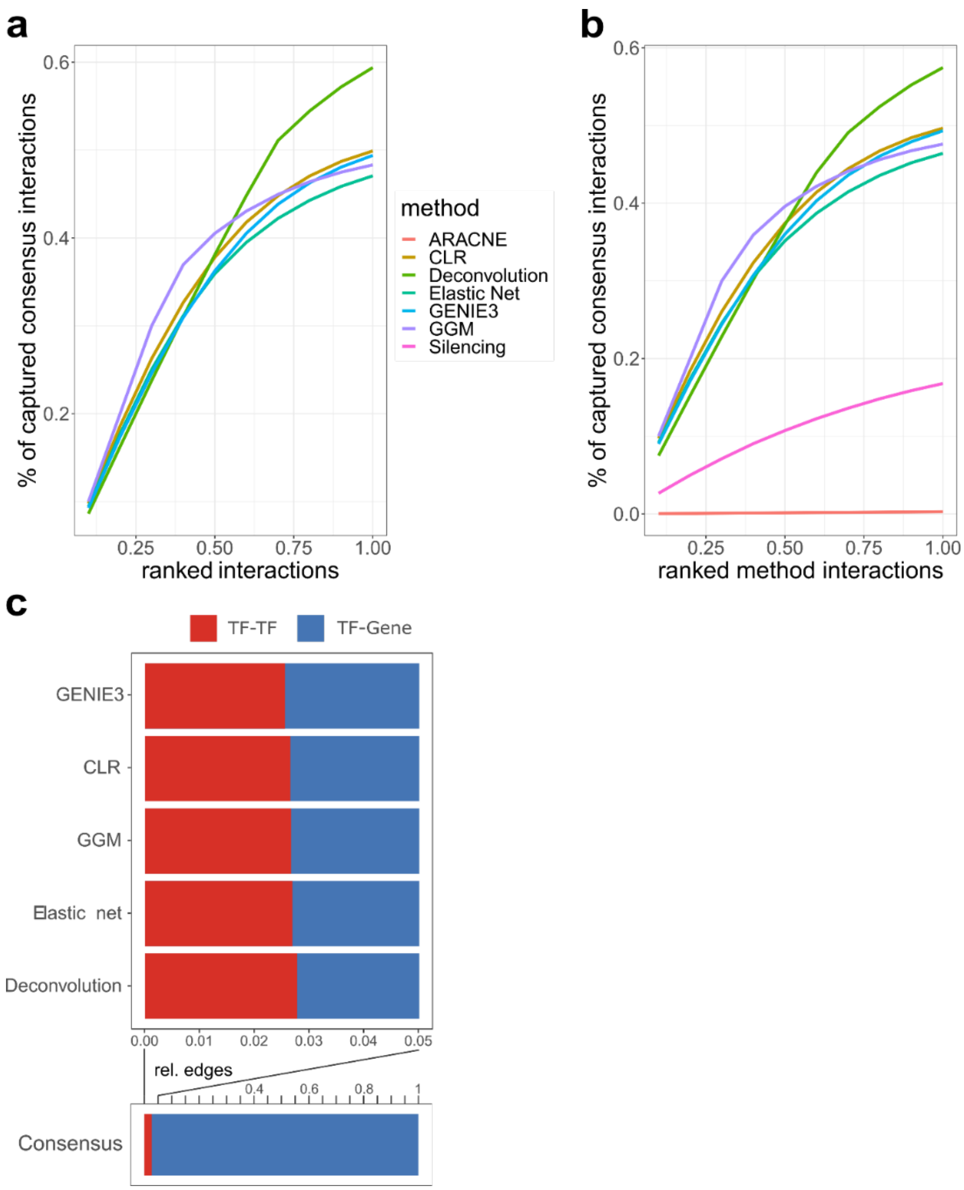

Supplementary Fig. 4: Overlap of consensus network with individual inference methods and proportion of TF-TF interactions in the obtained GRNs. a. The graph shows the overlap between the edges of the consensus network and the ranked interactions of the individual approaches normalized to the total number of edges in the consensus network used for regulator prediction. **b.** Same as in a but plotting the overlap with a consensus of all used approaches (including ARACNE and Silencing). **c**. Bar plots provide the proportion of TF-TF interactions (red) in comparison to TF-target gene interactions (blue) contained in the GRNs inferred by the different approaches and the consensus GRN resulting from their integration. All depicted analyses considered only the interactions within in the 10% network density threshold (see **Methods**).

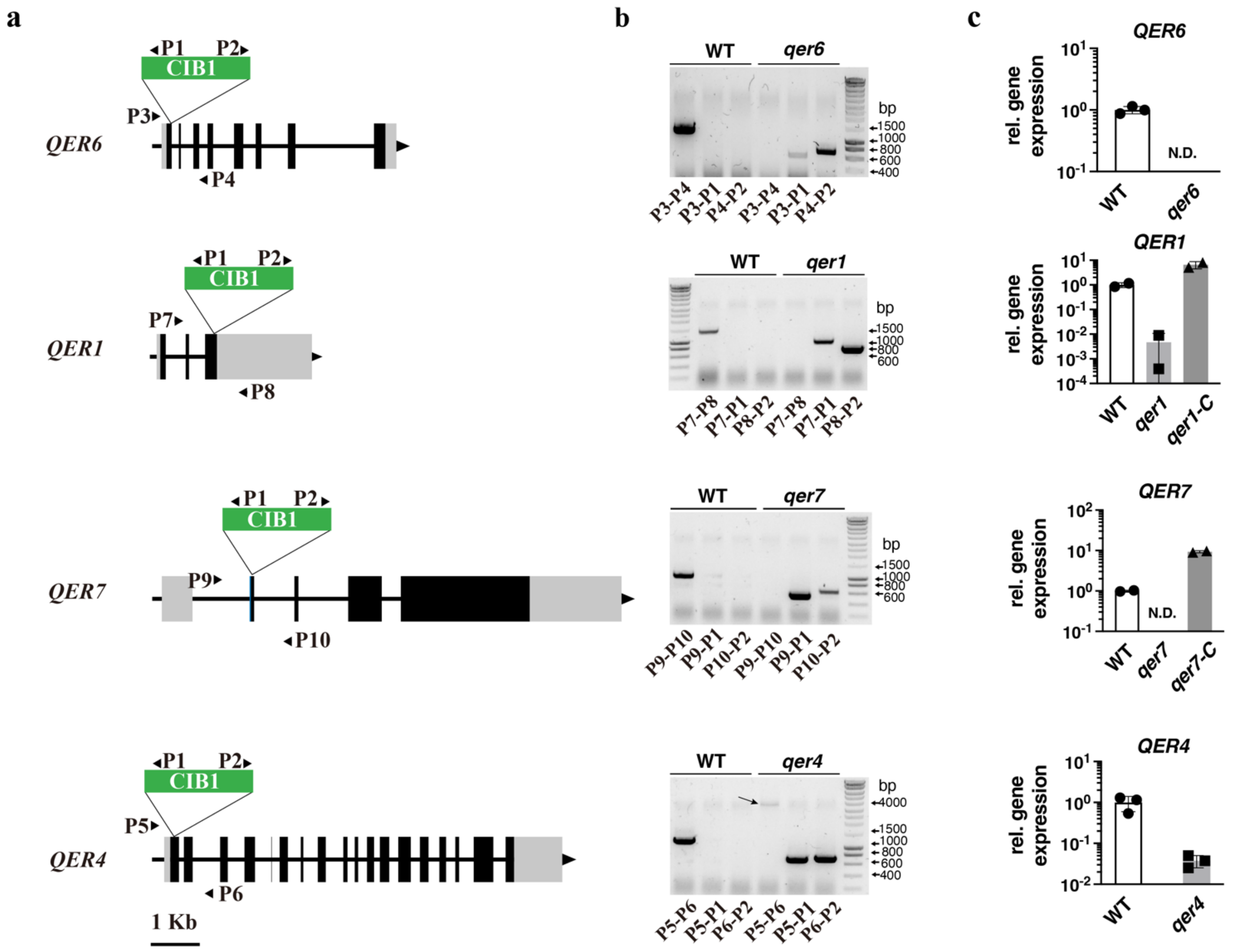

Supplementary Fig. 5: Genotyping of CLiP mutants affected in the predicted regulators of qE-related genes. **a**. Insertion map of the CIB1 cassette in the different genes. Exons are shown in black, introns as interconnecting lines, 5'UTR and 3'UTR in light gray, and primers in arrows. The insertion site of the CIB1 cassette is indicated by the triangle; **b**. PCR-validation of the insertion site in the different CLiP mutants using genomic DNA. To confirm the CIB1 insertion site, gene-specific primers were used that anneal upstream and downstream of the predicted insertion site of the cassette (primer pairs P3-P4, P7-P8, P9-P10 and P5-P6 for *qer6*, *qer1*, *qer7* and *qer4* respectively; **Supplementary Table 12**). Pairs of primers used are indicated at the bottom of the agarose gels used to separate the PCR products. Note that the PCR product of P5-P6 is indicated by an arrow. **c.** Relative expression levels of predicted qE regulator genes in the different CLiP mutants after exposure to HL (300 µmol photons m^{-2} s^{-1}) for 1h. For QER6 and QER4: n = 3 biological samples, mean ± sd; for QER1 and QER7: n = 2 biological samples.

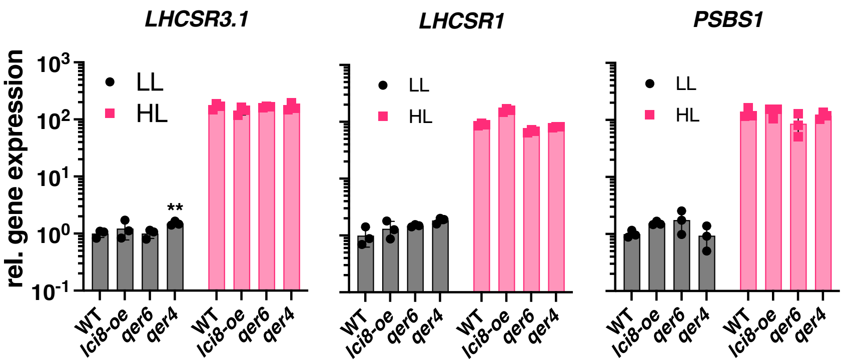

Supplementary Fig. 6: qE gene and protein expression in mutants bearing mutations in predicted qE regulator genes. WT, *lci8-oe, qer6* and *qer4* cells were acclimated for 16h in LL (15 µmol photons m^{-2} s^{-1}). After sampling for the LL conditions, light intensity was increased to 300 µmol photons m^{-2} s^{-1} (HL); samples were taken 1 h after exposure to HL. Shown are relative expression levels of *LHCSR3.1*, *LHCSR1* and *PSBS1* at the indicated conditions normalized to WT LL (*n* = 3 biological samples, mean ± sd). The two-sided p-values for the comparisons between the mutants and the WT are based on ANOVA Dunnett's multiple comparisons test on log10- transformed values and are indicated in the graphs (**, P = 0.0032).

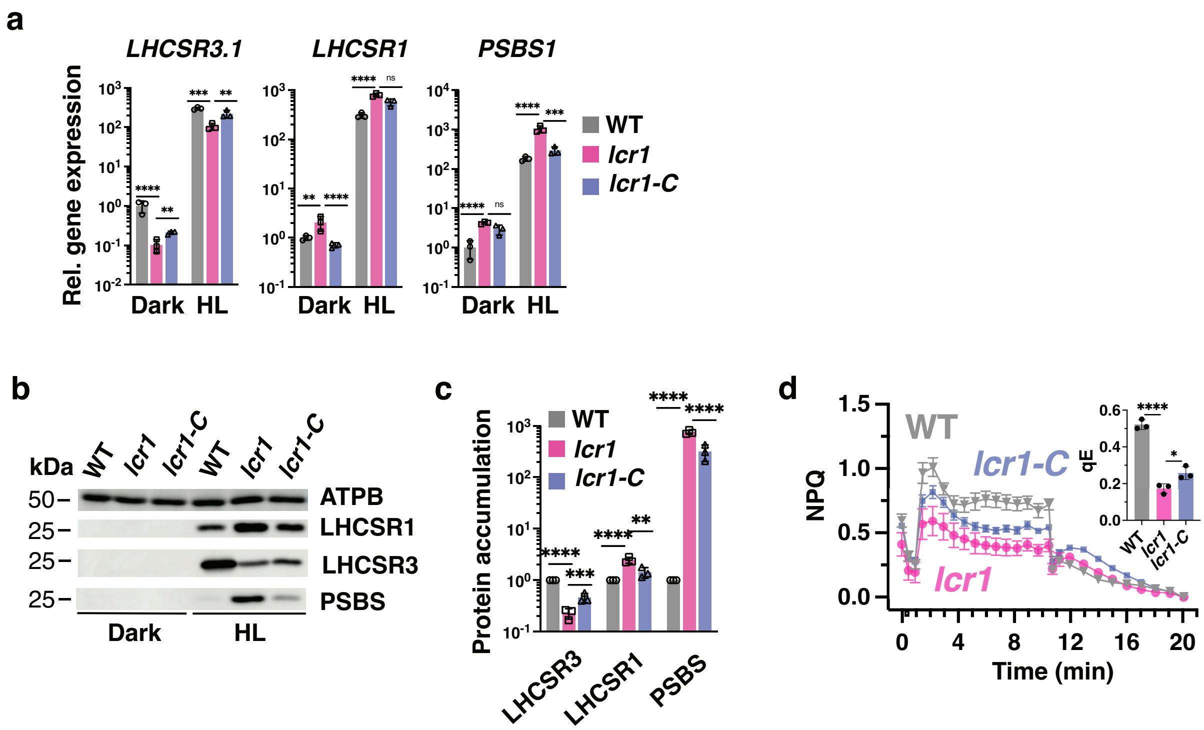

Supplementary Fig. 7: LCR1 is required for proper expression of qE-related genes during transitions from dark-to-light. WT, *lcr1* and *lcr1-C* cells were acclimated for 16h in darkness. After sampling for the dark conditions, light intensity was increased to 300 µmol photons m^{-2} s^{-1} (HL); samples were taken 1 h (RNA) or 4 h (protein and photosynthetic measurements) after exposure to HL. **a**. Relative expression levels of qE genes at the indicated conditions normalized to WT LL (n = 3 biological samples, mean ± sd). **b**. Immunoblot analyses of LHCSR1, LHCSR3, PSBS and ATPB (loading control) of one out of the three biological replicate samples, under the indicated conditions. **c.** Summary graph of immunoblots of all replicate samples of Supplementary Fig. 4b after normalization to ATPB. Shown are the HL treated samples; WT protein levels were set as 1 (n = 3 biological samples, mean ± sd). **d.** NPQ and calculated qE, 4h after exposure to HL (n = 3 biological samples, mean ± s.d). **a, c, d.** The two-sided p-values for the comparisons are based on ANOVA Dunnett's multiple comparisons test and are indicated in the graphs (*, P < 0.005, **, P < 0.01, ***, P < 0.001, ****, P < 0.0001). The exact p-values can be found in the Source Data file. Statistical analyses for panel **a** and **c** were applied on log10- transformed values.

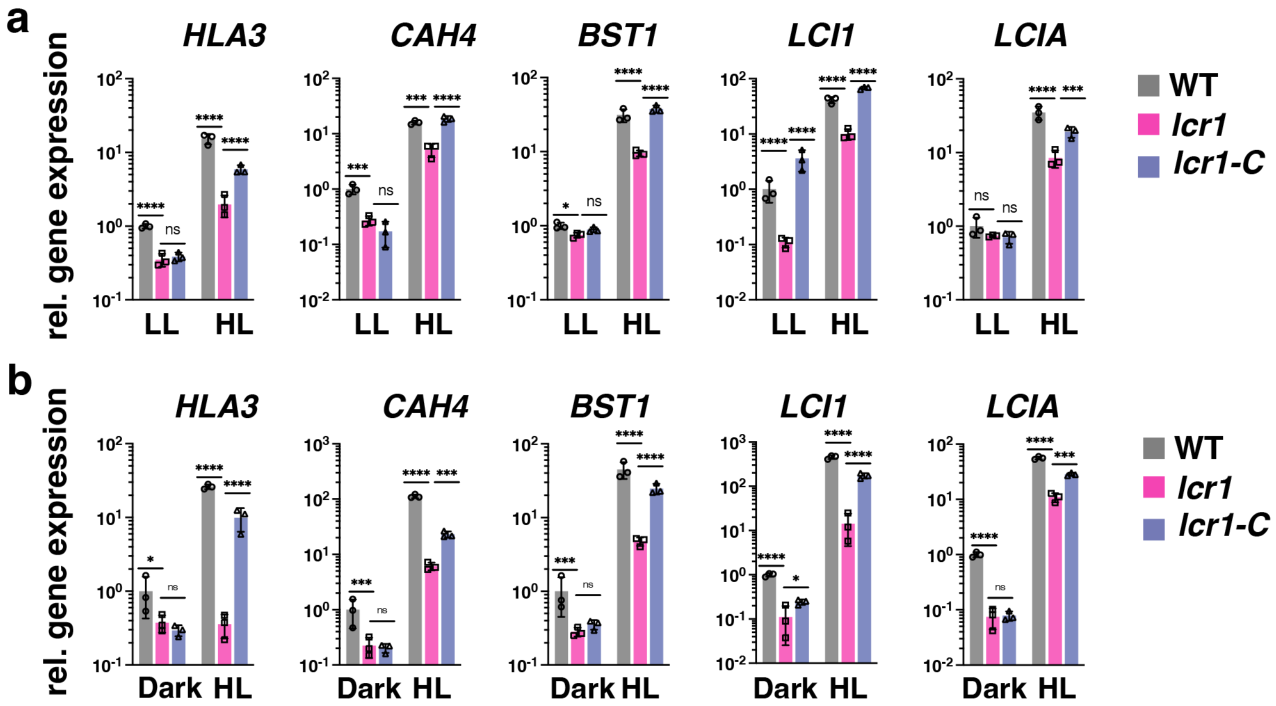

Supplementary Fig. 8: LCR1 activates the transcription of CCM genes in LL and dark-acclimated cells. a. WT, *lcr1* and *lcr1-C* cells were acclimated for 16h in LL (15 µmol photons m^{-2} s^{-1}). After sampling under LL conditions, light intensity was increased to 300 µmol photons m^{-2} s^{-1} (HL) and samples were taken 1 h after exposure to HL. Relative expression of CCM genes at the indicated conditions normalized to WT under LL (n = 3 biological samples, mean ± sd). **b**. Relative expression of CCM genes acclimated in 16h of darkness (n = 3 biological samples, mean ± sd). **a and b**. The two-sided p-values for the comparisons are based on ANOVA Dunnett's multiple comparisons test on log10- transformed values and are indicated in the graphs (*, $P < 0.005$, **, $P < 0.01$, ***, $P < 0.001$, ****, $P < 0.0001$). The exact p-values can be found in the Source Data file.

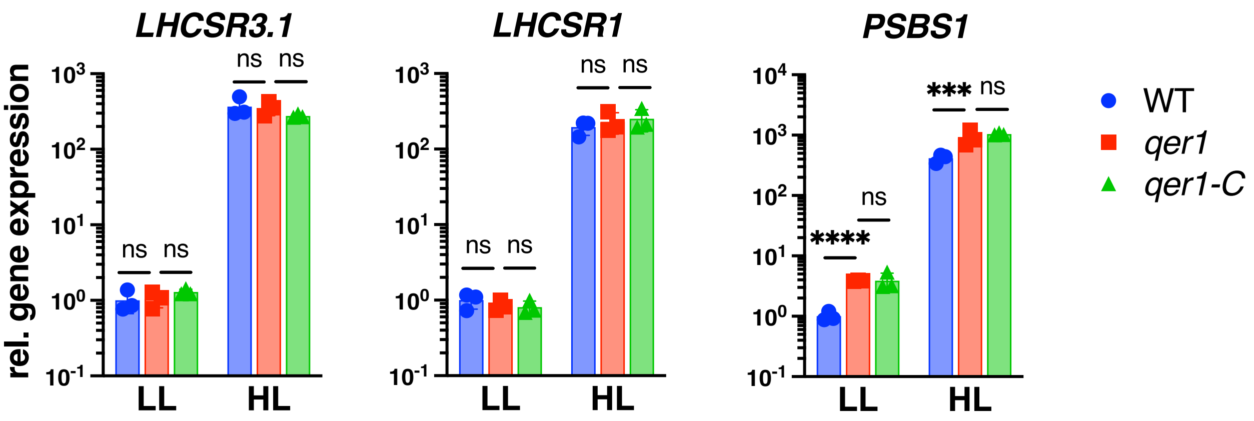

Supplementary Fig.9: Complementation of *qer1* with *QER1* (*qer1-C* strain) fails to rescue the *LHCSR1* and *PSBS* phenotypes. WT, *qer1* and *qer1-C* cells were acclimated for 16h in LL (15 µmol photons m^{-2} s^{-1}). After sampling for the dark conditions, light intensity was increased to 300 µmol photons m^{-2} s^{-1} (HL); RNA samples were taken 1 h after exposure to HL. Shown are relative expression levels of qE genes at the indicated conditions normalized to WT LL (*n* = 3 biological samples, mean ± sd). The two-sided p-values for the comparisons are based on ANOVA Dunnett's multiple comparisons test on log10- transformed values and are indicated in the graphs (***, P = 0.0008, ****, P < 0.0001).

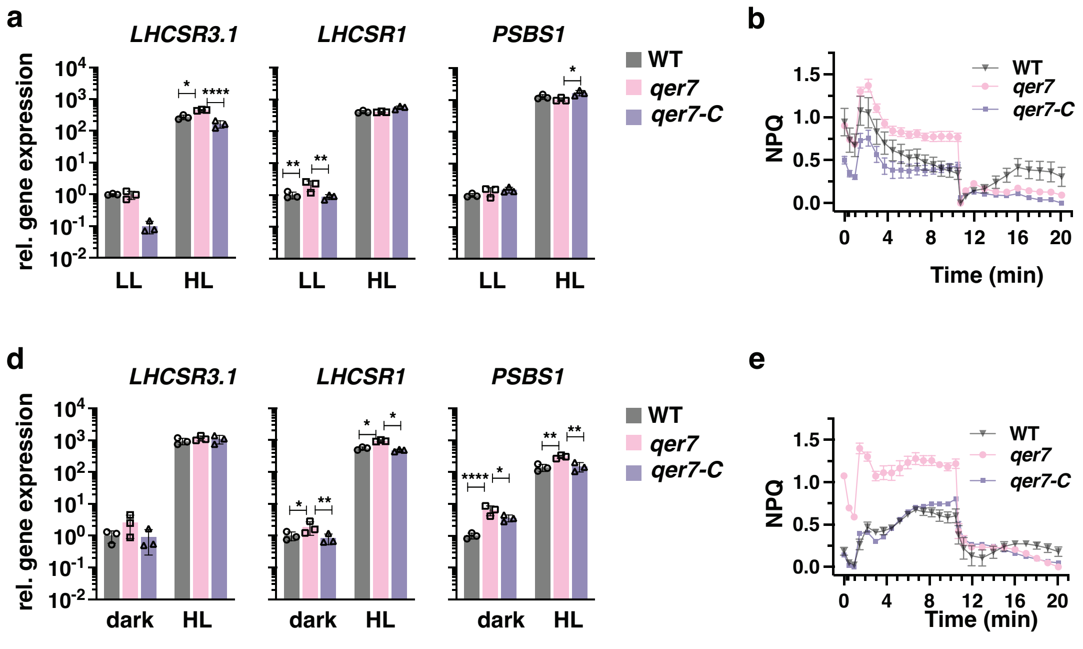

Supplementary Fig.10: Relative expression of qE-related genes in asynchronous *qer7* cells. WT, *qer7* and *qer7-C* cells were acclimated for 16h in LL (15 µmol photons m^{-2} s^{-1}; **a-c**) or darkness (**d-f**). After sampling for the LL or dark conditions, light intensity was increased to 300 µmol photons m^{-2} s^{-1} (HL) and samples were taken 1 h (RNA) or 4 h (photosynthetic measurements) after exposure to HL. **a, d**. Relative expression of qE and CCM genes at the indicated conditions normalized to WT LL (**a**) or dark (**d**) respectively (*n* = 3 biological samples, mean ± sd). **b, e.** NPQ and **c, f.** calculated qE, 4h after exposure to HL (*n* = 3 biological samples, mean ± s.d. **a, c, d, f.** The two-sided p-values for the comparisons are based on ANOVA Dunnett's multiple comparisons test and are indicated in the graphs (*, P < 0.005, **, P < 0.01, ***, P < 0.001, ****, P < 0.0001). Statistical analyses for panel **a** and **d** were applied on log_{10}- transformed values. The exact p-values can be found in the Source Data file.

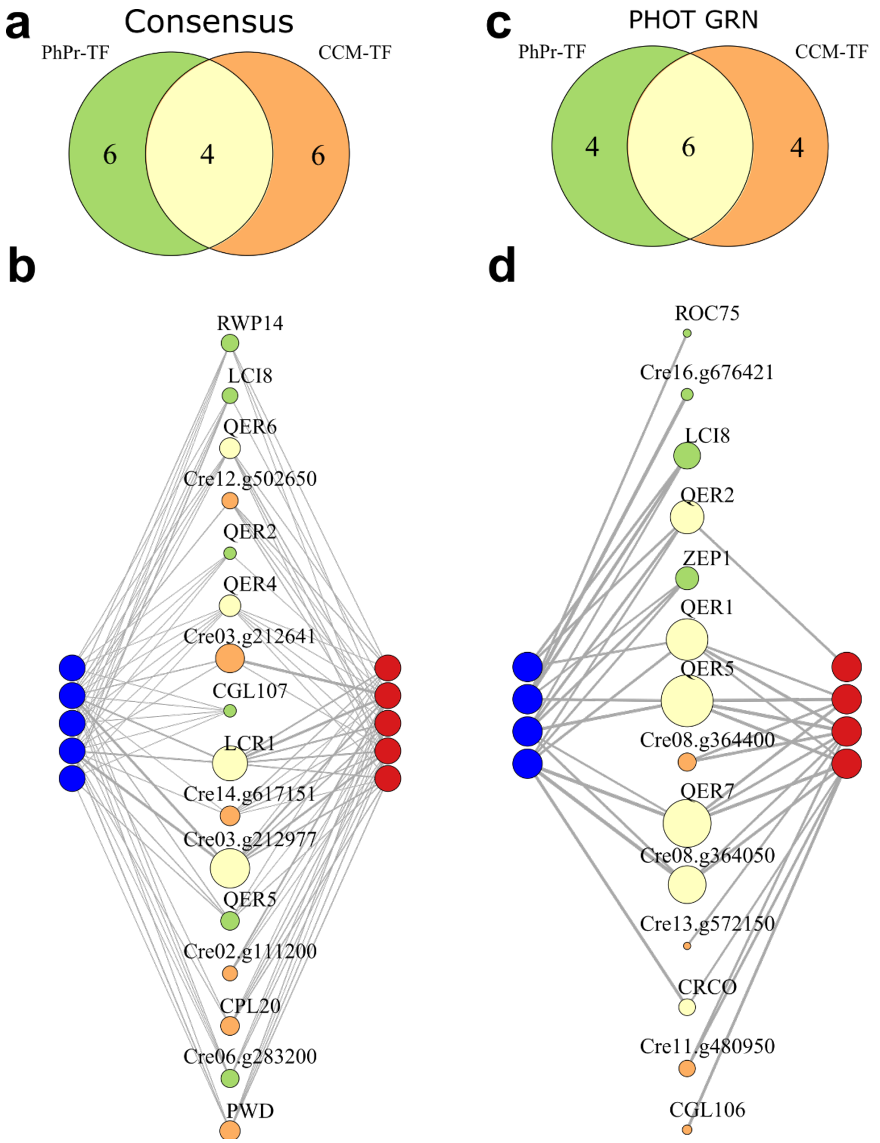

Supplementary Fig. 11: Reconstructed GRNs capture experimentally found regulators of qE and CCM. Top: Venn diagram depicting the overlap of the top 10 predicted regulators of carbon concentration mechanism (CCM) genes included in **Fig. 4a ,** and photoprotective (PhPr) genes based on **a.** the consensus or **c.** PHOT-specific GRN. Bottom: Network representation of the top ten TF sets of **b.** the consensus network or **d.** the phot GRN (center nodes, same color code as in panel **a**) and the target genes present in the respective network. qE-related genes (*LHCSR1*, *LHCSR3.1*, *LHCSR3.2*, *PSBS1*, *PSBS2*) are plotted in blue, and CCM genes included in **Fig. 4a** (*HLA3, CAH4, LCR1, BST1, LCI1*, *LCIA*) in red. In **b** the plotted regulatory strength corresponds to $\log(\frac{1}{r_{consen}})$, in **d.**, it corresponds to the GENIE3 edge weights denoting random forest importance measure. The edge width is proportional to the strength of the specific regulatory interaction. Size of the TF nodes corresponds to the sum of all plotted target gene edge weights. See also **Supplementary Table 4,6** for QER locus IDs.

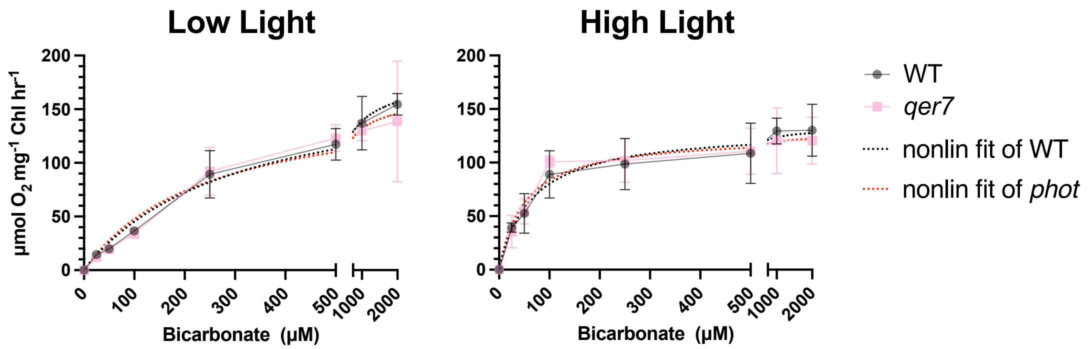

Supplementary Fig. 12: Ci affinity measurements in *qer7*. Oxygen evolution as a function of external Ci for WT and *qer7* cells grown under continuous LL (15 µmol m^{-2} s^{-1}) or exposed to HL (300 µmol m^{-2} s^{-1}) for 4h, in photoautotrophic conditions (Sueoka's high salt medium; HSM), at 23 °C in Erlenmeyer flasks shaken at 125 rpm (n = 3 biological samples, mean ± sd). Non-linear curve fitting to the Michaelis-Menten equation is shown in dotted lines. $k_{1/2}$(Ci) and Vmax values are presented in **Supplementary Table 7**.

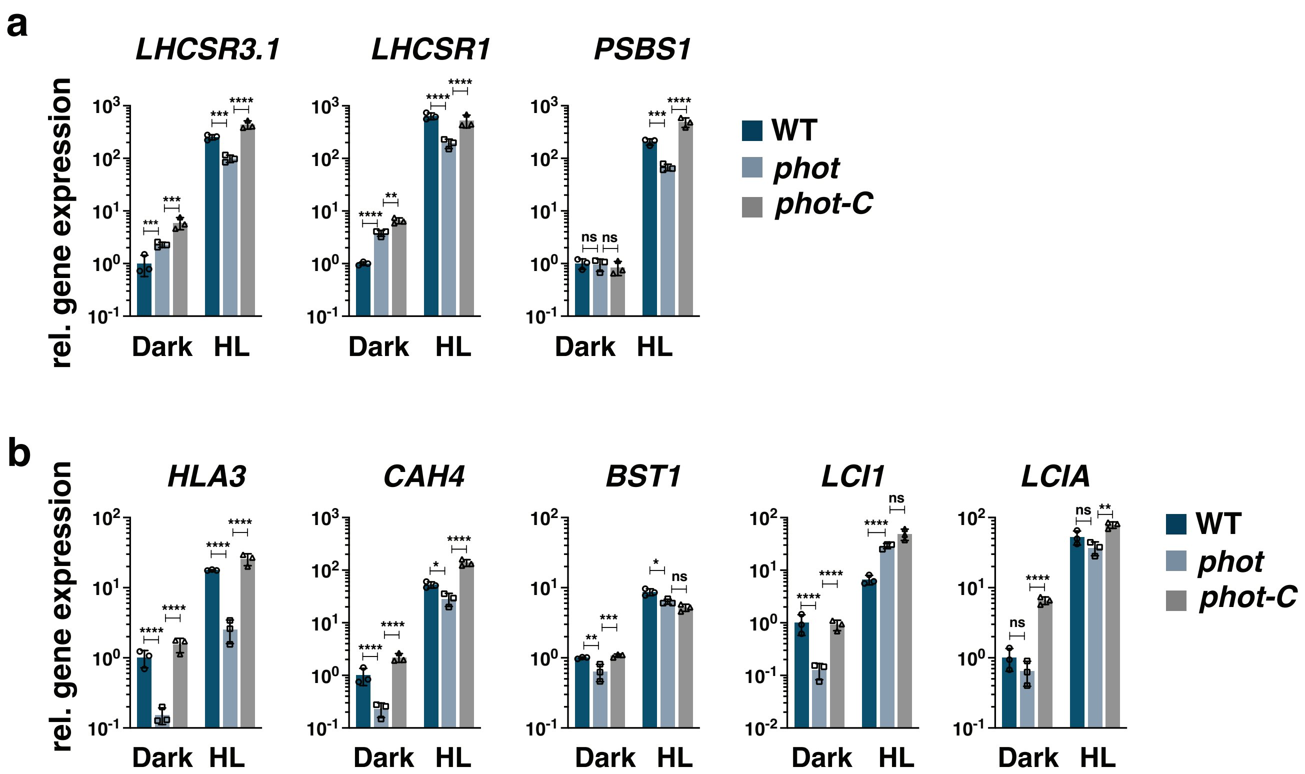

Supplementary Fig. 13: Phototropin dependent regulation of CCM gene expression. WT, *phot* and *phot-C* cells were synchronized under 12h light (15 µmol m^{-2} s^{-1})/12h dark cycles, in photoautotrophic conditions (Sueoka's high salt medium; HSM), at 23 °C in Erlenmeyer flasks shaken at 125 rpm. After sampling at the end of the dark phase, cells were exposed to 300 µmol photons m^{-2} s^{-1} (HL) and samples were taken 1 h after HL exposure. Relative expression levels of CCM (**a**) and qE (**b**) genes at the indicated conditions normalized to WT dark (*n* = 3 biological samples, mean ± sd). The two-sided p-values for the comparisons are based on ANOVA employing Dunnett's multiple comparisons test on log10-transformed values and are indicated in the graphs (*, P < 0.005, **, P < 0.01, ***, P < 0.001, ****, P < 0.0001). The exact p-values can be found in the Source Data file.

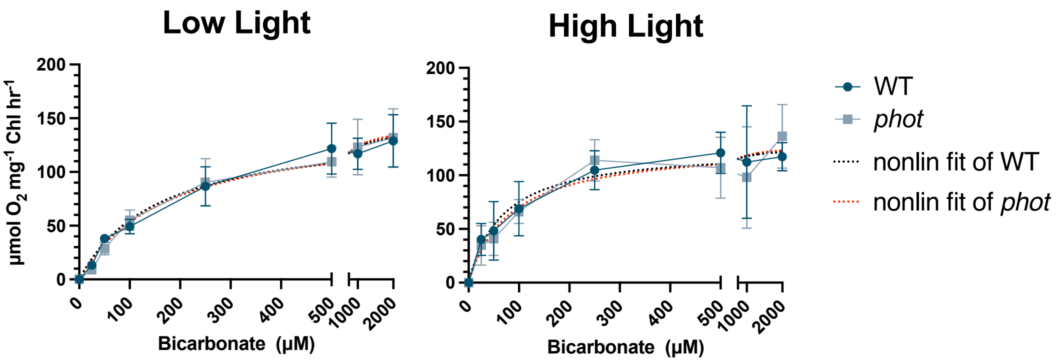

Supplementary Fig. 14: Oxygen evolution as a function of external Ci for WT and *phot* cells grown under continuous LL (15 µmol m^{-2} s^{-1}) or exposed to HL (300 µmol m^{-2} s^{-1}) for 4h, in photoautotrophic conditions (Sueoka's high salt medium; HSM), at 23 ^{o}C in Erlenmeyer flasks shaken at 125 rpm (*n* = 3 biological samples, mean ± sd). Non-linear curve fitting to the Michaelis-Menten equation is shown in dotted lines. $k_{1/2}$(Ci) and Vmax values are presented in **Supplementary Table 7**.

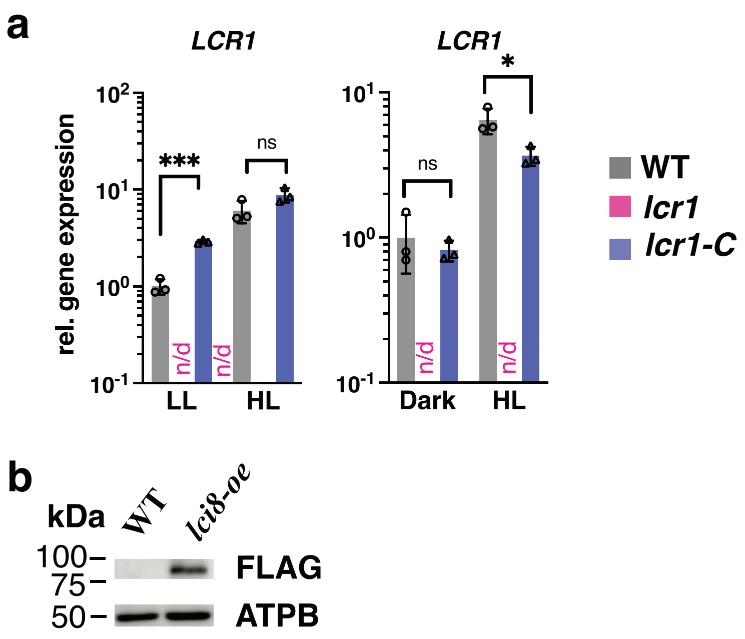

Supplementary Fig. 15: LCR1 mutation and LCI8 overexpression confirmation. a. Relative expression of *LCR1* in WT, *lcr1* and *lcr1-C* strains. Cells were acclimated for 16h in LL or darkness. After sampling for the LL or dark conditions, light intensity was increased to 300 µmol photons m^{-2} s^{-1} (HL), and samples for RNA purification were taken 1 h after exposure to HL (n = 3 biological samples, mean ± sd). *LCR1* gene expression was non-detectable (indicated as n/d in the graphs) in the *lcr1* mutant. The two-sided p-values for the comparisons are based on unpaired t-tests on log10- transformed values and are indicated in the graphs (*, $P < 0.005$, ***, $P < 0.001$). The exact p-values can be found in the Source Data file. **b.** Immunoblot analysis of LCI8-FLAG fused protein in WT and *lci8-oe* cells grown in TAP, LL. Representative immunoblot of an experiment repeated twice.

Supplementary Table Captions

Supplementary Table 1: RNAseq data sets used in this study.

Supplementary Table 2: List of Transcription factors used in this study; Family info was adapted from refs 41 and 42.

Supplementary Table 3: Edge list representation of the consensus network with mean ranks resulting from Borda count election method (refs 48 and 52) as edge attributes.

Supplementary Table 4: Top 10 predicted regulators of qE genes in the consensus GRN.

Supplementary Table 5: Edge list representation of the PHOT-specific GRN with importance score from GENIE 3 (ref. 68) as edge attributes.

Supplementary Table 6: Top 10 predicted regulators of qE genes in the PHOT-specific GRN.

Supplementary Table 7: Supplementary Table 7: $k_{1/2}$(Ci) and Vmax values calculated from External Data Fig. 12 and 14.

Supplementary Table 8: List of genes putatively involved in photoprotection used for regulator prediction.

Supplementary Table 9: List of genes putatively involved in CCM used for regulator prediction.

Supplementary Table 10: Global coregulators of photoprotection and CCM based on the consensus network.

Supplementary Table 11: Global coregulators of photoprotection and CCM based on the PHOT specific network.

Supplementary Table 12: PCR primers for CLiP mutant validation and complementation in this study.

Supplementary Table 13: RT-qPCR primers for all genes in this study.

Supplementary list of references

1. Li, X. *et al.* A genome-wide algal mutant library and functional screen identifies genes required for eukaryotic photosynthesis. *Nat. Genet.* **51,** 627–635 (2019).

2. Marbach, D. *et al.* Wisdom of crowds for robust gene network inference. *Nat. Methods* **9,** 796–804 (2012).
