# Supplementary material for: Widening the landscape of transcriptional regulation of green algal photoprotection
Source: Nat Commun. 2023 May 10;14:2687. doi: 10.1038/s41467-023-38183-4 (PMC10172295; doi:10.1038/s41467-023-38183-4)
Supplement: Supplementary file 3 — Description of Additional Supplementary Files [file 41467_2023_38183_MOESM3_ESM.pdf]

Description of Additional Supplementary Files

Supplementary Data 1: RNAseq data sets used in this study.

Supplementary Data 2: List of Transcription factors used in this study; Family info was adapted from refs 41 and 42.

Supplementary Data 3: Edge list representation of the consensus network with mean ranks resulting from Borda count election method (refs 48 and 52) as edge attributes.

Supplementary Data 4: Top 10 predicted regulators of qE genes in the consensus GRN.

Supplementary Data 5: Edge list representation of the PHOT-specific GRN with importance score from GENIE 3 (ref. 68) as edge attributes.

Supplementary Data 6: Top 10 predicted regulators of qE genes in the PHOT-specific GRN.

Supplementary Data 7: Supplementary Data 7: k1/2(Ci) and Vmax values calculated from External Data Fig.12 and 14.

Supplementary Data 8: List of genes putatively involved in photoprotection used for regulator prediction.

Supplementary Data 9: List of genes putatively involved in CCM used for regulator prediction.

Supplementary Data 10: Global coregulators of photoprotection and CCM based on the consensus network.

Supplementary Data 11: Global coregulators of photoprotection and CCM based on the PHOT specific network.

Supplementary Data 12: PCR primers for CLiP mutant validation and complementation in this study.

Supplementary Data 13: RT-qPCR primers for all genes in this study.
